# The Relationship between *Helicobacter pylori* and Beta-2 Microglobulin in Humans

**DOI:** 10.1155/2014/615089

**Published:** 2014-08-27

**Authors:** Abdullah Özgür Yeniova, Metin Kucukazman, Naim Ata, Kursat Dal, Ayşe Kefeli, Sebahat Başyiğit, Bora Aktaş, Kadir Okhan Akın, Yaşar Nazlıgül

**Affiliations:** ^1^Department of Gastroenterology, Kecioren Teaching and Research Hospital, Kecioren, 06380 Ankara, Turkey; ^2^Department of Internal Medicine, Kecioren Teaching and Research Hospital, Kecioren, 06380 Ankara, Turkey; ^3^Department of Clinical Biochemistry, Kecioren Teaching and Research Hospital, Kecioren, 06380 Ankara, Turkey

## Abstract

*H. pylori* is related to various gastrointestinal diseases. *β*_2_ Microglobulin (*β*_2_M) is an intrinsic element of major histocompatibility complex (MHC I). Serum *β*_2_M level may increase in inflammatory states. The aim of current study is to evaluate the relationship between *β*_2_M and* H. pylori* bearing CagA strains.* Methods*.* H. pylori* status was determined by histopathology of samples taken from stomach. CagA status and *β*_2_M level were measured from blood samples of patients. Eradication therapy was administered to the patients with* H. pylori* infection. *β*_2_ Microglobulin levels were measured before and after treatment.* Results*. 35 (29.2%)* H. pylori*(−) patients and 85 (70.8%)* H. pylori* (+) patients were included in the study. There were 52 (43.3%) patients with CagA negative and 33 (27.5%) patients with CagA positive* H. pylori* infection. The mean serum *β*_2_M level was 1.83 mg/L in* H. pylori* (−) group, 1.76 mg/L in* H. pylori* (+) CagA (−) group, and 1.93 mg/L in* H. pylori* and CagA (+) group (*P* > 0.05). Serum *β*_2_M levels (1.82 versus 1.64 mg/L *P* < 0.05) were decreased after eradication.* Conclusion*.* H. pylori* and CagA status did not affect *β*_2_M level. Relationship between low grade systematic inflammation and* H. pylori* should be investigated to find out new predictors for diseases associated with inflammation.

## 1. Introduction

Discovery of* Helicobacter pylori* (*H. pylori*) changed the management and treatment of many gastrointestinal diseases. Depending on the geographic location,* H. pylori* is an inhabitant of half of the world population's stomach.* H. pylori* plays a crucial role in the development of gastrointestinal diseases such as chronic gastritis, gastric ulcer and duodenal ulcer mucosa associated lymphoid tissue lymphoma (MALTOMA), and gastric adenocarcinoma [1, 2].

Despite its high prevalence, most of* H. pylori* infected subjects are asymptomatic. However some of them do not remain in gastritis stage and progress to gastric ulcer, duodenal ulcer, or gastric cancer [3]. Factors that contribute to disease progression have been recognised in the past decade. Cytotoxin-associated antigen (CagA) is one of the examples of* H. pylori* virulence factors that are encoded by genes of* H. pylori* “pathogenicity island” [4]. CagA is associated with significant inflammation in gastric mucosa.

*β*_2_-microglobulin (*β*_2_M) is the light chain domain of major histocompatibility complex (MHC I) [5]. It plays a crucial role in antigen presentation by chaperoning the assembly of the complex [6, 7]. *β*_2_M is synthesized by all nucleated cells but is released form lymphocytes mainly so it can be a marker for inflammation [8]. *β*_2_M is bound noncovalently, so it can be detected as free monomer in serum, urine, and cerebrospinal fluid. After degradation of MHC I molecules, *α* heavy chain remains in the cell but *β*_2_M is released into circulation. Therefore, *β*_2_M serum level is associated with cell turnover and renal function. Impaired glomerular function and increased cell turnover raise *β*_2_M levels. Elevated serum *β*_2_M level is poor prognosis of multiple myeloma, lymphomas, and leukemias [9–11].

We hypothesized that CagA positive* H. pylori* infection can be linked with increased levels of *β*_2_M. Relationship between *β*_2_M and* H. pylori* has been evaluated in the past decades [12–14] but CagA positivity in* H. pylori* infection and its association with *β*_2_M have not been evaluated.

The purpose of this study was to evaluate the relationship between *β*_2_M and CagA status of* H. pylori* infection. We also aimed to investigate the effect of eradication on *β*_2_M levels.

## 2. Material and Methods

The patients with dyspepsia who were admitted to the gastroenterology outpatient clinic between June 2012 and December 2012 were evaluated. Patients between 18 and 65 years of age who were referred to upper endoscopy from outpatient clinic were enrolled in the study. Patients who used antibiotics or nonsteroidal anti-inflammatory drugs for any reason in the past two weeks were excluded from the study. Local ethical committee approved the study protocol and informed consent was obtained from each enrolled patient.

Also all patients were evaluated for prior chronic diseases. Routine hemogram tests and biochemical parameters which include alanine amino transferase (ALT), aspartate amino transferase (AST), alkaline phosphatase (ALP), gamma glutamyl transpeptidase (GGT), total and direct bilirubin, urea, creatine, and serum electrolytes were performed for all participants. Patients with abnormal values and patients with coronary artery disease, liver failure, kidney failure, cerebrovascular disease, and malignant disease which may contribute to serum levels of *β*_2_M were also excluded from the study.

All patients who fulfilled the criteria underwent upper gastrointestinal tract endoscopy and patients who were taken biopsies from antrum and corpus were considered eligible for assessment. Other patients for whom biopsy material could not be obtained were also excluded from the study. We also excluded patients with gastric cancer and gastric and duodenal ulcer determined in endoscopy. Samples from antrum and corpus were transported in formalin contained solutions and were embedded in paraffin blocks. Four-micrometer-thick sections from each specimen were stained with hematoxylin-eosin for histopathologic evaluation and with Giemsa for* H. pylori* evaluation. Not only diagnosis of* H. pylori* infection but also assessment of intensity of* H. pylori*, inflammation, activation, atrophy, and intestinal metaplasia according to Sydney classification has been determined from the stained specimens. Pathologists who were blinded to patients reported the histopathological assessments as normal (0), mild (1), moderate (2), and severe (3) for each parameter of Sydney classification.

Fasting blood samples were obtained by the venipuncture of the large antecubital veins of the studied patients without stasis, after a 12-hour fast for measured *β*_2_M and* H. pylori* CagA status.

Comparison of serum levels of *β*_2_M before and after the eradication therapy was another aim of the current study. Participants who were diagnosed with* H. pylori* infection were administered* H. pylori* eradication therapy for 14 days. Eradication regimen was a combination of esomeprazole, 20 mg b.i.d., colloidal bismuth subcitrate, 600 mg b.i.d., tetracycline, 500 mg q.i.d., and metronidazole, 500 mg t.i.d. Six weeks after the last day of therapy, C14 urea-breath test (Heliprobe, Kibion AB Uppsala, Sweden) was performed for the patients.

Blood samples of patients who were negative for urea-breath test were collected. The samples were then centrifuged immediately; the plasma was separated and stored at −80°C. In order to avoid variation, all samples were studied on the same day and using the same kit. Commercial kit (DIA.PRO Diagnostic Bioprobes Srl, Milano, Italy) was used for analysis of* H. pylori* CagA status according to the manufacturer's instructions. *β*_2_M analysis was performed by using commercial kit (DiaSorin, Saluggia, Italy) according to the manufacturer's instructions.

SPSS version 15.0 statistical software was used for statistical analyses. Descriptive results were reported as mean ± standard deviation and median with minimum and maximum values. Results were analysed for normal distribution by using Kolmogorov-Smirnov. Student's *t*-test, Mann Whitney *U*, and Kruskal Wallis test were used where appropriate. Wilcoxon test was used to determine the comparison between serum levels of *β*_2_M before and after the eradication therapy. Correlation between serum levels of *β*_2_M and parameters of Sydney classification was determined by Spearman's correlation test. *P* value <0.05 was accepted as statistically significant.

## 3. Results

A total of 120 patients were included in the study. There were 68 (56.7%) female and 52 (43.4%) male patients.* H. pylori* infection was positive in 85 (70.8%) and negative in 35 (29.2%) patients. There were 52 (43.3%) patients who were CagA negative and 33 (27.5%) patients who were CagA positive in* H. pylori* infection positive group.

There was no statistically significant difference between* H. pylori* positive and negative (36.7 versus 37.2 resp.; *P* > 0.05) or CagA positive and negative (*P* > 0.05) subjects with regards to age and gender.

**Figure 1:**
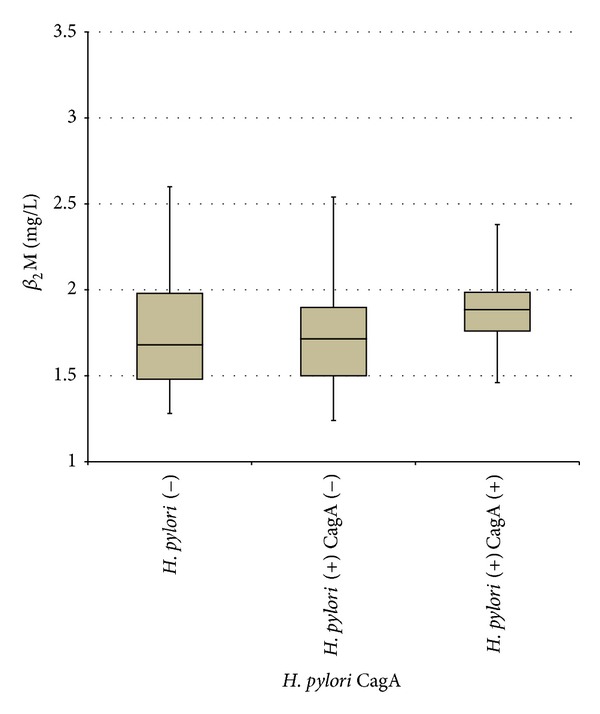
*β*_2_M level according to* H. pylori* and CagA status.

There was no significant difference in mean serum *β*_2_M levels (1.82 mg/L versus 1.83 mg/L resp.; *P* > 0.05) between patients with and without* H. pylori* infection. There was also no significant difference in mean serum *β*_2_M levels between* H. pylori* negative patients group,* H. pylori* positive-CagA negative group, and* H. pylori* positive-CagA positive group (1.83 mg/L versus 1.76 mg/L versus 1.93 mg/L, *P* > 0.05 resp.) (Figure 1).

Sydney classification parameters were evaluated in biopsy specimens which were conducted from antrum and corpus. The highest value of these parameters was used in correlation analysis of* H. pylori* intensity, acute and chronic inflammation, atrophy, intestinal metaplasia, and serum *β*_2_M level. There was no significant correlation between Sydney classification parameters and serum *β*_2_M levels (*P* > 0.05) (Table 1).

Eighty-five patients who were* H. pylori* positive were administered* H. pylori* eradication therapy. Ten patients were lost to followup for biopsy. Four patients had to give up the therapy due to nonspecific adverse effect of therapy. A total of 17 patients were lost to followup for urea-breath test control. Urea-breath test could be performed to 54 patients. Urea-breath test was positive in 4 and negative in 50 patients. Intent to treat ratio was 50/71 (70%) while 50/54 (92.5%) per protocol. Blood samples were obtained from 50 patients for *β*_2_M level after eradication therapy. *β*_2_M levels (1.82 versus 1.64 mg/L, resp.; *P* < 0.05) were significantly decreased after eradication therapy.

## 4. Discussion

The current study showed no association with* H. pylori* infection and serum *β*_2_M level. Although* H. pylori* infection causes inflammation of gastric mucosa, it has no effect on serum *β*_2_M levels.* H. pylori* infection with CagA strains did not have any effect on *β*_2_M level. Although patients with CagA positive* H. pylori* infection had the highest serum *β*_2_M levels, it was not statistically significant. Severity of gastric inflammation did not correlate with serum *β*_2_M; however, serum *β*_2_M levels were* significantly* decreased after* H. pylori* eradication.

*H. pylori* infection leads to accumulation of neutrophils and lymphocytes in the gastric mucosa. This accumulation progresses into chronic inflammation because* H. pylori* cannot be eradicated from mucosa by immune system. This local inflammation may trigger a systematic response in the host.* H. pylori* is suggested to be related with low grade inflammation [15]. This low grade inflammation regarding* H. pylori* infection is associated with diabetes mellitus and atherosclerosis [16, 17]. CagA is one of the most virulent factors of* H. pylori.* CagA positive* H. pylori* causes more serious gastric mucosa damage.* H. pylori* infection with CagA strains was associated with coronary artery diseases and cerebrovascular disease related with low grade inflammation more than CagA negative* H. pylori* infection [18, 19].

As serum *β*_2_M level reflects the activated lymphocytes, it is also considered as a marker of inflammation. There is growing evidence regarding the relationship between *β*_2_M and diseases associated with systemic inflammation. One study determined that elevated *β*_2_M levels reflect the severity of Crohn disease [20]. *β*_2_M levels were found to be elevated and correlated with disease severity in peripheral artery disease [21]. It is considered that hypoxia shed *β*_2_M from cell membranes. Also *β*_2_M level predicts cardiovascular mortality and all-cause mortality in general population, in population with renal impairment, and in elderly population [22–24]. Considering the association of *β*_2_M and inflammatory states, we hypothesized that *β*_2_M may increase in* H. pylori* infection, especially with CagA positive strains.

Previous studies have reported the same results on the relationship of serum *β*_2_M level and* H. pylori* infection. Our study had reiterated these previous results. First study in the literature was from pre-*H. pylori* era. In this study, serum *β*_2_M levels were measured in patients with malign and benign diseases of stomach. Elevated levels were determined in both groups of diseases but patients with gastric adenocarcinomas and gastric ulcer had elevated serum *β*_2_M levels more frequent than patients with gastritis and duodenal ulcer. But the difference was not statistically significant and* H. pylori* was not discovered yet when this study was published [12].

Two studies have reported that serum *β*_2_M levels have no relationship with* H. pylori* infection. Also correlation between serum *β*_2_M levels and* H. pylori* severity and intensity according to Sydney classification cannot be found. Akay et al. compared serum *β*_2_M levels of 30 patients with* H. pylori* and 22 patients without* H. pylori* infection. They failed to find a statistically significant difference in the mean of serum *β*_2_M levels between two groups.* H. pylori* intensity had increasing effect on serum *β*_2_M levels but it was not statistically significant [13].

Dincer et al. have conducted a study that aimed to find a correlation between serum *β*_2_M levels and parameters of Sydney classification for three regions of stomach (incisura angularis, antrum, and corpus). They were not able to find a correlation, but although it was not significant, biopsies from corpus showed that moderate and severe inflammation caused elevated serum *β*_2_M levels compared to mild inflammation [14].

While the previous reports did not determine difference in the mean of serum *β*_2_M levels, tissue concentrations of *β*_2_M did differ. In the same study, Conz et al. showed that 19 of 30 patients had subendothelial *β*_2_M but none of the control group had subendothelial *β*_2_M. Also one study conducted on uremic dialysis patients showed accumulation of *β*_2_M in gastric mucosa with* H. pylori* infection. Patients without* H. pylori* infection did not show this finding [25].

Our study had some limitations. Although the present study had the highest number of patients among the previous studies, the patient number was low. Tissue concentrations of *β*_2_M were not measured. These limitations may be a reason for the nonsignificant results.

## 5. Conclusion

To the best of our knowledge, this is the first report that investigated the association between *β*_2_M, CagA, and eradication therapy. Although there is no association, this possible relationship should be investigated by further studies with more patients. It is important to find reliable biomarkers for systemic inflammation and possible associations with chronic infections like* H. pylori* because diseases associated with systemic inflammations' severity and extension can be predicted with these biomarkers.

## Figures and Tables

**Table 1:** Correlation between *β*_2_M level and parameters of Sydney classification.

Parameters	*r*	*P*
HP	−0.039	0.671
Neutrophil	−0.036	0.695
Mononuclear cell	−0.009	0.922
Atrophy	0.003	0.973
Intestinal metaplasia	0.132	0.150
